# Supplementary material for: Proteomic Profiling of COVID-19 Patients Sera: Differential Expression with Varying Disease Stage and Potential Biomarkers
Source: Diagnostics (Basel). 2024 Nov 13;14(22):2533. doi: 10.3390/diagnostics14222533 (PMC11592820; doi:10.3390/diagnostics14222533)
Supplement: Supplementary file 1 [file diagnostics-14-02533-s001.zip › diagnostics-3272488-supplementary.pdf]

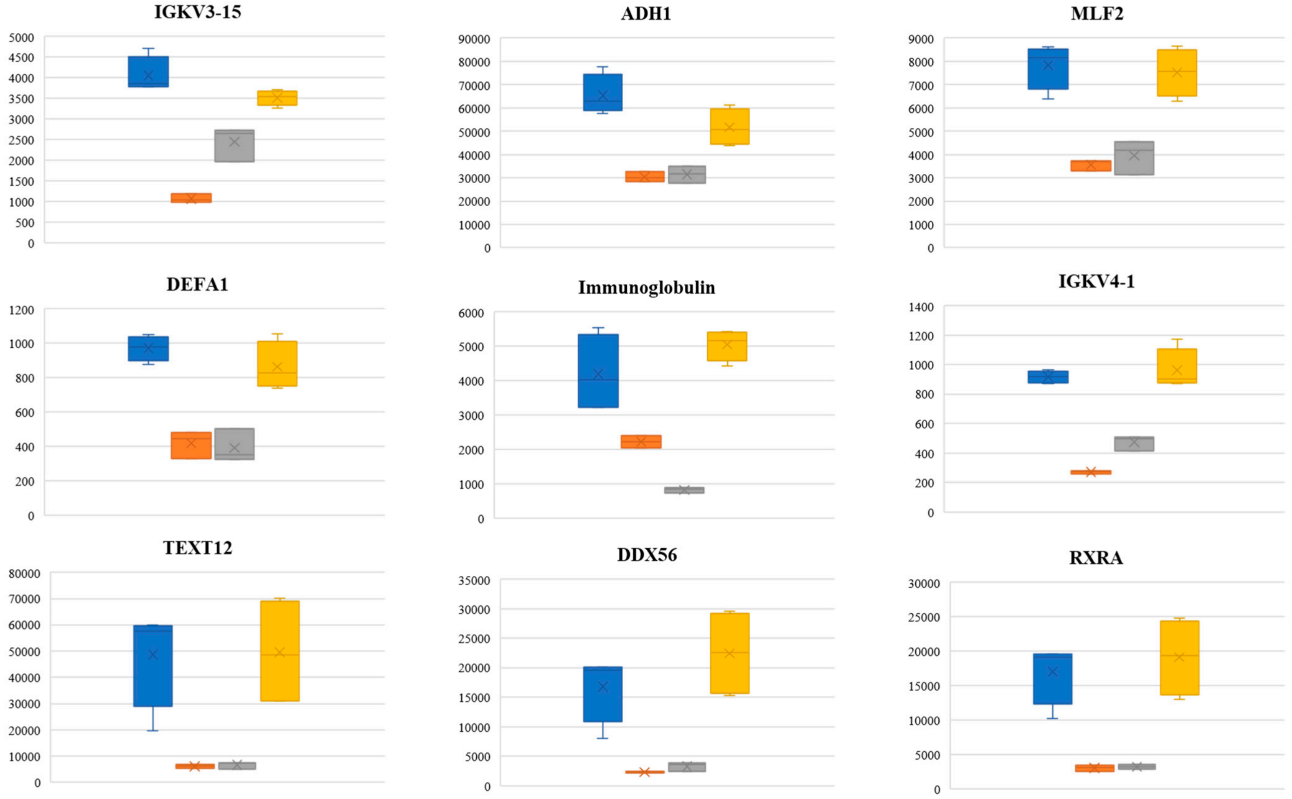

Figure S1. Box plot showing downregulated potential protein biomarkers in moderate and under medication patients. Blue represents healthy, Orange represents moderates, Grey represents under-medication, and Yellow represents recovered.

Table S1. Detailed list of the differentially 288 expressed proteins among the studied categories

Accession	Uniprot	Peptide count	Unique peptides	Confidence score	Anova (p)	q Value	Max fold change	Power	Highest mean condition	Lowest mean condition	Description
A0A0B4J1V0	IGHV3-15	3	1	37.391	1.22E-09	1.06E-09	3.798	1	PBP-CVD-HC22-03022022	PBP-CVD-U-Rx17-03022022	Immunoglobulin heavy variable 3-15
A0A0C4DH38	IGHV5-51	3	3	18.191	1.40E-08	5.65E-09	20.087	1	PBP-CVD-HC22-03022022	PBP-CVD-MOD15-03022022	Immunoglobulin heavy variable 5-51
A0A075B6I9	IGLV7-46	3	3	32.600	1.28E-11	3.31E-11	241.314	1	PBP-CVD-HC22-03022022	PBP-CVD-MOD15-03022022	Immunoglobulin lambda variable 7-46
A0A075B6N3	TRBV24-1	2	1	18.110	6.48E-04	5.57E-05	15.534	0.997	PBP-CVD-HC22-03022022	PBP-CVD-MOD15-03022022	T cell receptor beta variable 24-1
A0A075B6R2	IGHV4-4	4	1	42.420	1.44E-06	2.85E-07	6.790	1	PBP-CVD-HC22-03022022	PBP-CVD-MOD15-03022022	Immunoglobulin heavy variable 4-4
A6NKP2	SDR42E2	2	1	13.421	2.03E-03	1.56E-04	47.588	0.978	PBP-CVD-HC22-03022022	PBP-CVD-U-Rx17-03022022	Putative short-chain dehydrogenase/reductase family 42E member 2
A6NM62	LRRC53	3	2	17.235	2.15E-04	2.07E-05	3.238	0.999	PBP-CVD-HC22-03022022	PBP-CVD-REC66-03022022	Leucine-rich repeat-containing protein 53
B1AJZ9	FHAD1	7	5	44.239	2.08E-04	2.04E-05	2.288	1	PBP-CVD-HC22-03022022	PBP-CVD-MOD15-03022022	Forkhead-associated domain-containing protein 1
O00468	AGRN	4	2	29.095	4.85E-05	5.78E-06	2.586	1	PBP-CVD-HC22-03022022	PBP-CVD-REC66-03022022	Agrin
O14791	APOL1	6	4	49.859	5.53E-03	3.88E-04	6.959	0.934	PBP-CVD-HC22-03022022	PBP-CVD-MOD15-03022022	Apolipoprotein L1
O15068	MCF2L	3	2	16.750	3.95E-08	1.38E-08	49.816	1	PBP-CVD-HC22-03022022	PBP-CVD-U-Rx17-03022022	Guanine nucleotide exchange factor DBS
O41798	gag-pol	3	2	16.748	1.60E-08	5.83E-09	4.207	1	PBP-CVD-HC22-03022022	PBP-CVD-REC66-03022022	Gag-Pol polyprotein
O43683	BUB1	7	5	43.574	1.95E-02	1.24E-03	3.311	0.784	PBP-CVD-HC22-03022022	PBP-CVD-MOD15-03022022	Mitotic checkpoint serine/threonine-protein kinase BUB1

O43719	HTATSF1	8	7	54.381	1.81E-05	2.50E-06	2.388	1	PBP-CVD-HC22-03022022	PBP-CVD-U-Rx17-03022022	HIV Tat-specific factor 1
O43866	CD5L	17	16	187.628	6.77E-12	3.11E-11	10.832	1	PBP-CVD-HC22-03022022	PBP-CVD-MOD15-03022022	CD5 antigen-like
O95267	RASGRP1	4	3	24.050	1.50E-08	5.74E-09	4.193	1	PBP-CVD-HC22-03022022	PBP-CVD-REC66-03022022	RAS guanyl-releasing protein 1
O95445	APOM	12	9	78.861	1.05E-11	3.11E-11	2.973	1	PBP-CVD-HC22-03022022	PBP-CVD-MOD15-03022022	Apolipoprotein M
P0DOX6	Unknown	45	24	330.831	1.37E-12	1.60E-11	20.224	1	PBP-CVD-HC22-03022022	PBP-CVD-MOD15-03022022	Immunoglobulin mu heavy chain
P0DOY3	IGLC3	10	2	145.064	2.06E-09	1.46E-09	6.959	1	PBP-CVD-HC22-03022022	PBP-CVD-U-Rx17-03022022	Immunoglobulin lambda constant 3
P00330	ADH1	24	23	213.361	2.11E-05	2.85E-06	2.152	1	PBP-CVD-HC22-03022022	PBP-CVD-MOD15-03022022	(P00330) Alcohol dehydrogenase I (EC 1.1.1.1).
P00734	F2	39	38	483.701	4.59E-03	3.29E-04	2.421	0.913	PBP-CVD-HC22-03022022	PBP-CVD-REC66-03022022	Prothrombin
P00738	HP	56	35	641.991	2.42E-06	4.47E-07	5.442	1	PBP-CVD-HC22-03022022	PBP-CVD-U-Rx17-03022022	Haptoglobin
P00746	CFD	2	2	10.268	5.71E-03	3.99E-04	7.216	0.894	PBP-CVD-HC22-03022022	PBP-CVD-REC66-03022022	Complement factor D
P00748	F12	15	13	143.008	2.74E-07	6.95E-08	4.025	1	PBP-CVD-HC22-03022022	PBP-CVD-REC66-03022022	Coagulation factor XII
P01591	JCHAIN	11	11	93.287	5.80E-09	2.82E-09	4.715	1	PBP-CVD-HC22-03022022	PBP-CVD-MOD15-03022022	Immunoglobulin J chain
P01593	IGKV1D-33	2	1	16.360	6.18E-04	5.33E-05	16.570	0.998	PBP-CVD-HC22-03022022	PBP-CVD-U-Rx17-03022022	Immunoglobulin kappa variable 1D-33
P01619	IGKV3-20	4	1	28.482	1.25E-06	2.57E-07	3.675	1	PBP-CVD-HC22-03022022	PBP-CVD-U-Rx17-03022022	Immunoglobulin kappa variable 3-20
P01624	IGKV3-15	2	2	14.802	1.07E-07	3.32E-08	3.799	1	PBP-CVD-HC22-03022022	PBP-CVD-MOD15-03022022	Immunoglobulin kappa variable 3-15
P01699	IGLV1-44	2	1	21.503	3.76E-09	2.09E-09	81.882	1	PBP-CVD-HC22-03022022	PBP-CVD-MOD15-03022022	Immunoglobulin lambda variable 1-44
P01700	IGLV1-47	2	1	22.711	6.34E-11	1.23E-10	11.970	1	PBP-CVD-HC22-03022022	PBP-CVD-MOD15-03022022	Immunoglobulin lambda variable 1-47

P01780	IGHV3-7	7	4	63.930	1.15E-08	4.79E-09	3.626	1	PBP-CVD-HC22-03022022	PBP-CVD-U-Rx17-03022022	Immunoglobulin heavy variable 3-7
P01871	IGHM	31	10	408.780	1.07E-11	3.11E-11	12.242	1	PBP-CVD-HC22-03022022	PBP-CVD-MOD15-03022022	Immunoglobulin heavy constant mu
P01877	IGHA2	16	1	182.938	3.74E-03	2.71E-04	2.558	0.955	PBP-CVD-HC22-03022022	PBP-CVD-U-Rx17-03022022	Immunoglobulin heavy constant alpha 2
P02655	APOC2	4	4	52.830	4.94E-08	1.65E-08	6.164	1	PBP-CVD-HC22-03022022	PBP-CVD-MOD15-03022022	Apolipoprotein C-II
P02656	APOC3	11	8	92.903	3.11E-06	5.59E-07	5.480	1	PBP-CVD-HC22-03022022	PBP-CVD-MOD15-03022022	Apolipoprotein C-III
P02671	FGA	6	3	48.966	3.05E-10	4.07E-10	10.235	1	PBP-CVD-HC22-03022022	PBP-CVD-REC66-03022022	Fibrinogen alpha chain
P02743	APCS	5	5	33.070	4.09E-05	4.98E-06	4.102	1	PBP-CVD-HC22-03022022	PBP-CVD-MOD15-03022022	Serum amyloid P-component
P02745	C1QA	5	5	39.418	1.37E-06	2.75E-07	3.286	1	PBP-CVD-HC22-03022022	PBP-CVD-MOD15-03022022	Complement C1q subcomponent subunit A
P02746	C1QB	12	10	91.701	2.67E-05	3.49E-06	2.649	1	PBP-CVD-HC22-03022022	PBP-CVD-MOD15-03022022	Complement C1q subcomponent subunit B
P02747	C1QC	6	4	51.155	2.22E-07	5.83E-08	4.395	1	PBP-CVD-HC22-03022022	PBP-CVD-MOD15-03022022	Complement C1q subcomponent subunit C
P02749	APOH	29	26	276.618	4.15E-05	5.02E-06	2.417	1	PBP-CVD-HC22-03022022	PBP-CVD-REC66-03022022	Beta-2-glycoprotein 1
P02753	RBP4	9	8	93.900	2.65E-07	6.81E-08	2.255	1	PBP-CVD-HC22-03022022	PBP-CVD-REC66-03022022	Retinol-binding protein 4
P02765	AHSG	16	14	178.762	1.25E-03	9.86E-05	2.729	0.991	PBP-CVD-HC22-03022022	PBP-CVD-MOD15-03022022	Alpha-2-HS-glycoprotein
P02766	TTR	2	2	24.755	2.96E-04	2.77E-05	3.366	0.998	PBP-CVD-HC22-03022022	PBP-CVD-REC66-03022022	Transthyretin
P02768	ALB	200	184	1244.891	5.85E-05	6.90E-06	3.033	1	PBP-CVD-HC22-03022022	PBP-CVD-U-Rx17-03022022	Serum albumin
P04278	SHBG	5	5	30.500	5.52E-06	8.96E-07	4.490	1	PBP-CVD-HC22-03022022	PBP-CVD-U-Rx17-03022022	Sex hormone-binding globulin
P05156	CFI	26	23	210.261	1.07E-04	1.15E-05	4.518	1	PBP-CVD-HC22-03022022	PBP-CVD-REC66-03022022	Complement factor I

P06727	APOA4	39	37	478.619	2.10E-04	2.05E-05	3.214	0.999	PBP-CVD-HC22-03022022	PBP-CVD-REC66-03022022	Apolipoprotein A-IV
P11137	MAP2	5	4	34.428	3.04E-04	2.82E-05	3.745	0.999	PBP-CVD-HC22-03022022	PBP-CVD-MOD15-03022022	Microtubule-associated protein 2
P18428	LBP	7	7	62.615	2.14E-03	1.63E-04	6.657	0.977	PBP-CVD-HC22-03022022	PBP-CVD-U-Rx17-03022022	Lipopolysaccharide-binding protein
P20851	C4BPB	6	5	40.031	7.57E-05	8.55E-06	2.522	1	PBP-CVD-HC22-03022022	PBP-CVD-U-Rx17-03022022	C4b-binding protein beta chain
P23142	FBLN1	7	7	42.647	1.02E-04	1.10E-05	2.734	1	PBP-CVD-HC22-03022022	PBP-CVD-U-Rx17-03022022	Fibulin-1
P28702	RXRB	2	1	12.612	1.21E-03	9.59E-05	8.283	0.995	PBP-CVD-HC22-03022022	PBP-CVD-MOD15-03022022	Retinoic acid receptor RXR-beta
P29622	SERPINA4	15	15	120.504	9.30E-05	1.02E-05	2.347	1	PBP-CVD-HC22-03022022	PBP-CVD-REC66-03022022	Kallistatin
P35542	SAA4	7	7	71.179	1.63E-07	4.65E-08	6.385	1	PBP-CVD-HC22-03022022	PBP-CVD-U-Rx17-03022022	Serum amyloid A-4 protein
P35900	KRT20	2	1	22.461	1.17E-04	1.23E-05	3.012	1	PBP-CVD-HC22-03022022	PBP-CVD-U-Rx17-03022022	Keratin_ type I cytoskeletal 20
P43652	AFM	31	26	285.382	1.75E-04	1.77E-05	2.371	1	PBP-CVD-HC22-03022022	PBP-CVD-REC66-03022022	Afamin
P48681	NES	8	7	60.079	5.02E-07	1.16E-07	7.280	1	PBP-CVD-HC22-03022022	PBP-CVD-MOD15-03022022	Nestin
P55056	APOC4	6	4	62.923	2.22E-11	5.18E-11	9.790	1	PBP-CVD-HC22-03022022	PBP-CVD-REC66-03022022	Apolipoprotein C-IV
P59665	DEFA1	3	2	23.529	3.22E-05	4.07E-06	2.484	1	PBP-CVD-HC22-03022022	PBP-CVD-U-Rx17-03022022	Neutrophil defensin 1
P61006	RAB8A	2	2	16.660	4.35E-04	3.90E-05	26.553	0.999	PBP-CVD-HC22-03022022	PBP-CVD-U-Rx17-03022022	Ras-related protein Rab-8A
P69905	HBA1	17	14	167.346	1.00E-04	1.09E-05	2.796	1	PBP-CVD-HC22-03022022	PBP-CVD-U-Rx17-03022022	Hemoglobin subunit alpha
Q5TBE3	C9orf153	3	2	18.278	3.15E-02	1.93E-03	3.479	0.718	PBP-CVD-HC22-03022022	PBP-CVD-MOD15-03022022	Uncharacterized protein C9orf153
Q5XXA6	ANO1	4	2	22.524	2.41E-04	2.29E-05	8.505	0.999	PBP-CVD-HC22-03022022	PBP-CVD-MOD15-03022022	Anoctamin-1
Q6ZN16	MAP3K15	14	11	81.258	2.90E-03	2.14E-04	4.035	0.966	PBP-CVD-HC22-03022022	PBP-CVD-U-Rx17-03022022	Mitogen-activated protein kinase kinase kinase 15
Q7L523	RRAGA	4	3	37.727	2.83E-07	7.02E-08	2.254	1	PBP-CVD-HC22-03022022	PBP-CVD-MOD15-03022022	Ras-related GTP-binding protein A

Q7Z6R9	TFAP2D	2	2	10.825	1.55E-08	5.76E-09	Infinity	1	PBP-CVD-HC22-03022022	PBP-CVD-MOD15-03022022	Transcription factor AP-2-delta
Q8N3J2	METTL4	2	2	17.731	1.33E-06	2.69E-07	3.226	1	PBP-CVD-HC22-03022022	PBP-CVD-MOD15-03022022	Methyltransferase-like protein 4
Q8NA23	WDR31	3	2	18.207	4.72E-03	3.36E-04	11.564	0.923	PBP-CVD-HC22-03022022	PBP-CVD-REC66-03022022	WD repeat-containing protein 31
Q8TD43	TRPM4	5	4	29.008	1.31E-07	3.90E-08	4.777	1	PBP-CVD-HC22-03022022	PBP-CVD-MOD15-03022022	Transient receptor potential cation channel subfamily M member 4
Q8WXE0	CASKIN2	5	3	33.058	6.15E-05	7.15E-06	3.423	1	PBP-CVD-HC22-03022022	PBP-CVD-U-Rx17-03022022	Caskin-2
Q9BT92	TCHP	6	4	49.363	9.78E-05	1.07E-05	19.715	1	PBP-CVD-HC22-03022022	PBP-CVD-MOD15-03022022	Trichoplein keratin filament-binding protein
Q9BXK1	KLF16	2	2	15.414	6.43E-03	4.48E-04	2.653	0.878	PBP-CVD-HC22-03022022	PBP-CVD-REC66-03022022	Krueppel-like factor 16
Q9BYX4	IFIH1	6	3	38.874	6.32E-06	1.01E-06	4.448	1	PBP-CVD-HC22-03022022	PBP-CVD-REC66-03022022	Interferon-induced helicase C domain-containing protein 1
Q9C091	GREB1L	5	2	33.671	1.04E-08	4.42E-09	7.190	1	PBP-CVD-HC22-03022022	PBP-CVD-U-Rx17-03022022	GREB1-like protein
Q9H3Q1	CDC42EP4	4	3	21.066	3.77E-06	6.67E-07	4.485	1	PBP-CVD-HC22-03022022	PBP-CVD-MOD15-03022022	Cdc42 effector protein 4
Q9H8W3	FAM204A	2	2	10.828	9.01E-04	7.47E-05	12.701	0.995	PBP-CVD-HC22-03022022	PBP-CVD-U-Rx17-03022022	Protein FAM204A
Q9H078	CLPB	2	1	13.618	1.16E-02	7.73E-04	3.332	0.815	PBP-CVD-HC22-03022022	PBP-CVD-REC66-03022022	Caseinolytic peptidase B protein homolog
Q9NQ66	PLCB1	5	2	26.057	4.02E-04	3.63E-05	15.356	0.999	PBP-CVD-HC22-03022022	PBP-CVD-U-Rx17-03022022	1-phosphatidylinositol 4_5-bisphosphate phosphodiesterase beta-1
Q9NR80	ARHGEF4	2	1	12.843	5.42E-10	5.76E-10	198.118	1	PBP-CVD-HC22-03022022	PBP-CVD-U-Rx17-03022022	Rho guanine nucleotide exchange factor 4

Q9NWL6	ASNSD1	3	2	17.996	4.90E-04	4.29E-05	2.404	0.998	PBP-CVD-HC22-03022022	PBP-CVD-U-Rx17-03022022	Asparagine synthetase domain-containing protein 1
Q9NZP8	C1RL	6	3	69.840	2.64E-03	1.97E-04	25.753	0.961	PBP-CVD-HC22-03022022	PBP-CVD-U-Rx17-03022022	Complement C1r subcomponent-like protein
Q9P2S6	ANKMY1	11	9	71.669	2.22E-05	2.95E-06	2.924	1	PBP-CVD-HC22-03022022	PBP-CVD-MOD15-03022022	Ankyrin repeat and MYND domain-containing protein 1
Q9UG01	IFT172	8	6	58.657	7.99E-09	3.66E-09	5.895	1	PBP-CVD-HC22-03022022	PBP-CVD-MOD15-03022022	Intraflagellar transport protein 172 homolog
Q9UKL3	CASP8AP2	6	4	40.098	3.77E-02	2.27E-03	3.453	0.679	PBP-CVD-HC22-03022022	PBP-CVD-U-Rx17-03022022	CASP8-associated protein 2
Q70Z35	PREX2	4	1	25.805	7.41E-03	5.08E-04	3.079	0.894	PBP-CVD-HC22-03022022	PBP-CVD-U-Rx17-03022022	Phosphatidylinositol 3_4_5 trisphosphate-dependent Rac exchanger 2 protein
Q86X45	DNAAF11	2	1	18.404	2.04E-04	2.03E-05	868.461	1	PBP-CVD-HC22-03022022	PBP-CVD-MOD15-03022022	Protein tilB homolog
Q96HU1	SGSM3	3	2	18.078	7.40E-05	8.43E-06	3.410	1	PBP-CVD-HC22-03022022	PBP-CVD-U-Rx17-03022022	Small G protein signaling modulator 3
Q96M83	CCDC7	5	3	33.499	1.83E-07	5.02E-08	5.334	1	PBP-CVD-HC22-03022022	PBP-CVD-REC66-03022022	Coiled-coil domain-containing protein 7
Q96Q89	KIF20B	6	4	39.826	8.81E-06	1.35E-06	7.189	1	PBP-CVD-HC22-03022022	PBP-CVD-MOD15-03022022	Kinesin-like protein KIF20B
Q03495	NSP HEV G2	3	1	17.587	8.62E-07	1.83E-07	6.029	1	PBP-CVD-HC22-03022022	PBP-CVD-U-Rx17-03022022	Non-structural polyprotein pORF1
Q14141	SEPTIN6	4	1	22.364	1.32E-03	1.03E-04	3.613	0.989	PBP-CVD-HC22-03022022	PBP-CVD-U-Rx17-03022022	Septin-6
Q14520	HABP2	11	10	80.647	1.94E-07	5.28E-08	2.706	1	PBP-CVD-HC22-03022022	PBP-CVD-MOD15-03022022	Hyaluronan-binding protein 2
Q14896	MYBPC3	5	1	36.877	1.59E-07	4.60E-08	29.072	1	PBP-CVD-HC22-03022022	PBP-CVD-U-Rx17-03022022	Myosin-binding protein C_ cardiac-type

Q15582	TGFBI	7	5	35.072	2.08E-04	2.04E-05	2.974	1	PBP-CVD-HC22-03022022	PBP-CVD-U-Rx17-03022022	Transforming growth factor-beta-induced protein ig-h3
Q15773	MLF2	2	2	15.672	2.88E-05	3.71E-06	2.193	1	PBP-CVD-HC22-03022022	PBP-CVD-MOD15-03022022	Myeloid leukemia factor 2
Q92954	PRG4	9	9	61.537	4.61E-06	7.92E-07	3.123	1	PBP-CVD-HC22-03022022	PBP-CVD-U-Rx17-03022022	Proteoglycan 4
Q99848	EBNA1BP2	2	2	10.988	9.59E-03	6.41E-04	6.637	0.849	PBP-CVD-HC22-03022022	PBP-CVD-U-Rx17-03022022	Probable rRNA-processing protein EBP2
A0A0B4J2D9	IGKV1D-13	3	2	32.269	1.17E-10	1.95E-10	31.976	1	PBP-CVD-MOD15-03022022	PBP-CVD-HC22-03022022	Immunoglobulin kappa variable 1D-13
A0A075B6J9	IGLV2-18	2	2	17.833	9.66E-06	1.45E-06	3.542	1	PBP-CVD-MOD15-03022022	PBP-CVD-U-Rx17-03022022	Immunoglobulin lambda variable 2-18
O60488	ACSL4	4	2	21.149	2.54E-04	2.40E-05	2.196	0.999	PBP-CVD-MOD15-03022022	PBP-CVD-HC22-03022022	Long-chain-fatty-acid--CoA ligase 4
O60496	DOK2	3	3	21.617	2.82E-08	9.98E-09	14.972	1	PBP-CVD-MOD15-03022022	PBP-CVD-HC22-03022022	Docking protein 2
O75636	FCN3	7	7	46.224	9.92E-05	1.08E-05	3.200	1	PBP-CVD-MOD15-03022022	PBP-CVD-HC22-03022022	Ficolin-3
O95425	SVIL	2	1	11.975	1.05E-03	8.48E-05	9.652	0.997	PBP-CVD-MOD15-03022022	PBP-CVD-U-Rx17-03022022	Supervillin
P0DJI8	SAA1	13	7	178.698	1.52E-08	5.74E-09	9.943	1	PBP-CVD-MOD15-03022022	PBP-CVD-U-Rx17-03022022	Serum amyloid A-1 protein
P0DJI9	SAA2	12	6	149.875	1.47E-08	5.71E-09	12.339	1	PBP-CVD-MOD15-03022022	PBP-CVD-HC22-03022022	Serum amyloid A-2 protein
P01009	SERPINA1	75	66	622.426	2.06E-10	3.01E-10	3.564	1	PBP-CVD-MOD15-03022022	PBP-CVD-U-Rx17-03022022	Alpha-1-antitrypsin
P01019	AGT	16	15	155.882	2.73E-04	2.56E-05	2.055	0.999	PBP-CVD-MOD15-03022022	PBP-CVD-REC66-03022022	Angiotensinogen
P01023	A2M	139	121	1367.449	5.70E-07	1.28E-07	2.899	1	PBP-CVD-MOD15-03022022	PBP-CVD-REC66-03022022	Alpha-2-macroglobulin
P01743	IGHV1-46	2	1	17.930	5.55E-04	4.82E-05	3.203	0.997	PBP-CVD-MOD15-03022022	PBP-CVD-HC22-03022022	Immunoglobulin heavy variable 1-46
P02741	CRP	5	5	44.902	6.01E-07	1.33E-07	45.447	1	PBP-CVD-MOD15-03022022	PBP-CVD-HC22-03022022	C-reactive protein

P02748	C9	26	24	246.246	2.03E-05	2.76E-06	2.704	1	PBP-CVD-MOD15-03022022	PBP-CVD-HC22-03022022	Complement component C9
P02750	LRG1	19	19	195.820	2.43E-06	4.47E-07	4.288	1	PBP-CVD-MOD15-03022022	PBP-CVD-U-Rx17-03022022	Leucine-rich alpha-2-glycoprotein
P02776	PF4	5	5	52.016	1.80E-05	2.50E-06	2.117	1	PBP-CVD-MOD15-03022022	PBP-CVD-U-Rx17-03022022	Platelet factor 4
P05543	SERPINA7	7	6	38.630	3.05E-05	3.90E-06	25.487	1	PBP-CVD-MOD15-03022022	PBP-CVD-HC22-03022022	Thyroxine-binding globulin
P05883	env	5	4	32.200	4.39E-10	4.89E-10	7.565	1	PBP-CVD-MOD15-03022022	PBP-CVD-HC22-03022022	Envelope glycoprotein gp160
P06681	C2	28	21	179.079	3.63E-05	4.49E-06	2.148	1	PBP-CVD-MOD15-03022022	PBP-CVD-REC66-03022022	Complement C2
P07360	C8G	7	7	61.066	1.34E-02	8.80E-04	2.245	0.837	PBP-CVD-MOD15-03022022	PBP-CVD-HC22-03022022	Complement component C8 gamma chain
P07996	THBS1	30	28	199.056	7.70E-09	3.60E-09	10.180	1	PBP-CVD-MOD15-03022022	PBP-CVD-U-Rx17-03022022	Thrombospondin-1
P08910	ABHD2	4	2	23.279	1.26E-04	1.31E-05	34.231	1	PBP-CVD-MOD15-03022022	PBP-CVD-HC22-03022022	Monoacylglycerol lipase ABHD2
P10643	C7	28	26	216.827	2.31E-04	2.21E-05	2.270	1	PBP-CVD-MOD15-03022022	PBP-CVD-U-Rx17-03022022	Complement component C7
P10809	HSPD1	2	1	11.206	8.36E-03	5.65E-04	16.422	0.896	PBP-CVD-MOD15-03022022	PBP-CVD-REC66-03022022	60 kDa heat shock protein_ mitochondrial
P11177	PDHB	3	3	23.578	1.81E-04	1.82E-05	5.931	1	PBP-CVD-MOD15-03022022	PBP-CVD-HC22-03022022	Pyruvate dehydrogenase E1 component subunit beta_mitochondrial
P13671	C6	42	39	353.075	3.77E-06	6.67E-07	3.986	1	PBP-CVD-MOD15-03022022	PBP-CVD-HC22-03022022	Complement component C6
P15135	E3 15.3 kDa	2	2	16.109	1.86E-02	1.19E-03	4.216	0.804	PBP-CVD-MOD15-03022022	PBP-CVD-HC22-03022022	Early E3 15.3 kDa protein
P15169	CPN1	8	8	66.363	2.15E-03	1.63E-04	2.145	0.989	PBP-CVD-MOD15-03022022	PBP-CVD-U-Rx17-03022022	Carboxypeptidase N catalytic chain
P15814	IGLL1	3	3	16.555	1.40E-05	2.00E-06	5.146	1	PBP-CVD-MOD15-03022022	PBP-CVD-HC22-03022022	Immunoglobulin lambda-like polypeptide 1
P20742	PZP	38	27	444.568	2.20E-06	4.20E-07	6.218	1	PBP-CVD-MOD15-03022022	PBP-CVD-HC22-03022022	Pregnancy zone protein

P30740	SERPINB1	3	2	17.366	4.96E-06	8.27E-07	3.809	1	PBP-CVD-MOD15-03022022	PBP-CVD-REC66-03022022	Leukocyte elastase inhibitor
P36980	CFHR2	10	5	73.543	9.44E-04	7.76E-05	3.039	0.990	PBP-CVD-MOD15-03022022	PBP-CVD-REC66-03022022	Complement factor H-related protein 2
P42356	PI4KA	2	1	12.550	6.16E-06	9.86E-07	4.805	1	PBP-CVD-MOD15-03022022	PBP-CVD-HC22-03022022	Phosphatidylinositol 4-kinase alpha
P43403	ZAP70	3	3	16.590	2.66E-09	1.68E-09	342.091	1	PBP-CVD-MOD15-03022022	PBP-CVD-HC22-03022022	Tyrosine-protein kinase ZAP-70
P48740	MASP1	5	5	26.610	2.44E-07	6.34E-08	10.216	1	PBP-CVD-MOD15-03022022	PBP-CVD-HC22-03022022	Mannan-binding lectin serine protease 1
P51884	LUM	11	8	81.223	3.66E-05	4.50E-06	2.369	1	PBP-CVD-MOD15-03022022	PBP-CVD-HC22-03022022	Lumican
P60709	ACTB	10	6	80.599	4.21E-07	9.84E-08	10.030	1	PBP-CVD-MOD15-03022022	PBP-CVD-HC22-03022022	Actin_ cytoplasmic 1
P61129	ZC3H6	6	4	43.489	2.99E-06	5.42E-07	9.740	1	PBP-CVD-MOD15-03022022	PBP-CVD-HC22-03022022	Zinc finger CCCH domain-containing protein 6
P69891	HBG1	3	2	33.579	2.26E-06	4.25E-07	6.870	1	PBP-CVD-MOD15-03022022	PBP-CVD-HC22-03022022	Hemoglobin subunit gamma-1
Q2M389	WASHC4	5	3	27.622	1.46E-04	1.50E-05	2.247	1	PBP-CVD-MOD15-03022022	PBP-CVD-HC22-03022022	WASH complex subunit 4
Q5TCY1	TTBK1	6	3	32.511	8.00E-04	6.70E-05	2.270	0.998	PBP-CVD-MOD15-03022022	PBP-CVD-U-Rx17-03022022	Tau-tubulin kinase 1
Q5TD97	FHL5	4	3	21.763	1.11E-03	8.91E-05	7.624	0.997	PBP-CVD-MOD15-03022022	PBP-CVD-U-Rx17-03022022	Four and a half LIM domains protein 5
Q5TEA3	DNAAF9	5	4	28.573	7.93E-05	8.91E-06	2.912	1	PBP-CVD-MOD15-03022022	PBP-CVD-HC22-03022022	Uncharacterized protein C20orf194
Q6P2S7	TTC41P	3	2	21.407	1.65E-04	1.68E-05	3.012	1	PBP-CVD-MOD15-03022022	PBP-CVD-REC66-03022022	Putative tetratricopeptide repeat protein 41
Q6P587	FAHD1	2	1	13.048	2.81E-07	7.02E-08	3.325	1	PBP-CVD-MOD15-03022022	PBP-CVD-HC22-03022022	Acylpyruvase FAHD1_ mitochondrial
Q6S8J3	POTEE	20	12	147.465	9.08E-09	4.01E-09	9.070	1	PBP-CVD-MOD15-03022022	PBP-CVD-HC22-03022022	POTE ankyrin domain family member E

Q8IV77	CNGA4	3	2	21.636	9.54E-04	7.82E-05	8.165	0.993	PBP-CVD-MOD15-03022022	PBP-CVD-HC22-03022022	Cyclic nucleotide-gated cation channel alpha-4
Q8IVL8	CPO	2	2	23.990	1.11E-04	1.18E-05	2.062	1	PBP-CVD-MOD15-03022022	PBP-CVD-HC22-03022022	Carboxypeptidase O
Q9H1K0	RBSN	5	3	35.237	7.75E-12	3.11E-11	19.981	1	PBP-CVD-MOD15-03022022	PBP-CVD-HC22-03022022	Rabenosyn-5
Q9H6S0	YTHDC2	4	3	22.121	1.32E-07	3.90E-08	19.512	1	PBP-CVD-MOD15-03022022	PBP-CVD-HC22-03022022	3'-5' RNA helicase YTHDC2
Q9HAW4	CLSPN	8	5	44.595	1.16E-07	3.58E-08	3.805	1	PBP-CVD-MOD15-03022022	PBP-CVD-U-Rx17-03022022	Claspin
Q9NX58	LYAR	3	2	23.960	1.36E-05	1.97E-06	24.237	1	PBP-CVD-MOD15-03022022	PBP-CVD-HC22-03022022	Cell growth-regulating nucleolar protein
Q9P2M7	CGN	11	9	52.992	1.93E-03	1.49E-04	2.029	0.984	PBP-CVD-MOD15-03022022	PBP-CVD-REC66-03022022	Cingulin
Q9UNY4	TTF2	4	3	28.005	1.22E-05	1.79E-06	9.010	1	PBP-CVD-MOD15-03022022	PBP-CVD-HC22-03022022	Transcription termination factor 2
Q9Y2K3	MYH15	6	3	34.841	3.94E-02	2.37E-03	3.030	0.667	PBP-CVD-MOD15-03022022	PBP-CVD-HC22-03022022	Myosin-15
Q9Y575	ASB3	2	1	12.310	1.57E-05	2.22E-06	493.218	1	PBP-CVD-MOD15-03022022	PBP-CVD-REC66-03022022	Ankyrin repeat and SOCS box protein 3
Q86VH2	KIF27	6	3	32.702	3.90E-06	6.86E-07	3.525	1	PBP-CVD-MOD15-03022022	PBP-CVD-HC22-03022022	Kinesin-like protein KIF27
Q86YT6	MIB1	4	3	22.592	1.20E-05	1.77E-06	11.644	1	PBP-CVD-MOD15-03022022	PBP-CVD-HC22-03022022	E3 ubiquitin-protein ligase MIB1
Q96C45	ULK4	4	2	22.535	2.18E-09	1.50E-09	46.475	1	PBP-CVD-MOD15-03022022	PBP-CVD-HC22-03022022	Serine/threonine-protein kinase ULK4
Q96KN2	CNDP1	9	8	50.825	2.12E-04	2.06E-05	2.058	1	PBP-CVD-MOD15-03022022	PBP-CVD-HC22-03022022	Beta-Ala-His dipeptidase
Q96KS9	FAM167A	3	2	15.748	3.18E-09	1.84E-09	5.024	1	PBP-CVD-MOD15-03022022	PBP-CVD-HC22-03022022	Protein FAM167A
Q96L93	KIF16B	2	1	19.130	3.15E-03	2.31E-04	3.272	0.950	PBP-CVD-MOD15-03022022	PBP-CVD-HC22-03022022	Kinesin-like protein KIF16B
Q03591	CFHR1	14	4	102.169	1.94E-10	3.01E-10	6.197	1	PBP-CVD-MOD15-03022022	PBP-CVD-HC22-03022022	Complement factor H-related protein 1

Q06033	ITIH3	27	26	223.025	6.75E-09	3.22E-09	4.423	1	PBP-CVD-MOD15-03022022	PBP-CVD-HC22-03022022	Inter-alpha-trypsin inhibitor heavy chain H3
Q06787	FMR1	4	3	28.013	2.15E-03	1.63E-04	2.298	0.982	PBP-CVD-MOD15-03022022	PBP-CVD-HC22-03022022	Synaptic functional regulator FMR1
Q08380	LGALS3BP	17	16	151.064	2.85E-05	3.71E-06	3.594	1	PBP-CVD-MOD15-03022022	PBP-CVD-HC22-03022022	Galectin-3-binding protein
Q13188	STK3	3	2	37.772	7.26E-06	1.14E-06	4.340	1	PBP-CVD-MOD15-03022022	PBP-CVD-HC22-03022022	Serine/threonine-protein kinase 3
Q13790	APOF	6	6	44.885	6.45E-03	4.49E-04	2.201	0.927	PBP-CVD-MOD15-03022022	PBP-CVD-HC22-03022022	Apolipoprotein F
Q14568	HSP90AA2P	3	3	15.637	1.97E-04	1.97E-05	9.389	0.999	PBP-CVD-MOD15-03022022	PBP-CVD-REC66-03022022	Heat shock protein HSP 90-alpha A2
Q14624	ITIH4	44	41	434.209	2.71E-02	1.69E-03	2.120	0.797	PBP-CVD-MOD15-03022022	PBP-CVD-U-Rx17-03022022	Inter-alpha-trypsin inhibitor heavy chain H4
A0A0B4J1U7	IGHV6-1	5	5	20.198	2.09E-07	5.61E-08	2.863	1	PBP-CVD-REC66-03022022	PBP-CVD-U-Rx17-03022022	Immunoglobulin heavy variable 6-1
A0A0B4J2H0	IGHV1-69D	3	1	18.910	5.83E-08	1.84E-08	13.362	1	PBP-CVD-REC66-03022022	PBP-CVD-MOD15-03022022	Immunoglobulin heavy variable 1-69D
A0A0C4DH29	IGHV1-3	2	1	11.865	5.75E-08	1.84E-08	3.354	1	PBP-CVD-REC66-03022022	PBP-CVD-U-Rx17-03022022	Immunoglobulin heavy variable 1-3
A0A0C4DH68	IGKV2-24	2	1	18.423	4.12E-03	2.97E-04	5.679	0.952	PBP-CVD-REC66-03022022	PBP-CVD-MOD15-03022022	Immunoglobulin kappa variable 2-24
A0A0C4DH73	IGKV1-12	3	1	25.719	2.23E-05	2.95E-06	215.194	1	PBP-CVD-REC66-03022022	PBP-CVD-MOD15-03022022	Immunoglobulin kappa variable 1-12
A0A075B6K0	IGLV3-16	2	1	14.224	1.83E-07	5.02E-08	8.668	1	PBP-CVD-REC66-03022022	PBP-CVD-MOD15-03022022	Immunoglobulin lambda variable 3-16
A0A075B6K4	IGLV3-10	2	1	22.106	9.66E-04	7.89E-05	2.225	0.994	PBP-CVD-REC66-03022022	PBP-CVD-U-Rx17-03022022	Immunoglobulin lambda variable 3-10
O43150	ASAP2	4	2	16.602	5.37E-03	3.79E-04	2.165	0.927	PBP-CVD-REC66-03022022	PBP-CVD-U-Rx17-03022022	Arf-GAP with SH3 domain_ ANK repeat and PH domain-containing protein 2
O60343	TBC1D4	3	1	23.913	3.34E-13	7.81E-12	173.595	1	PBP-CVD-REC66-03022022	PBP-CVD-HC22-03022022	TBC1 domain family member 4

O75037	KIF21B	10	7	71.835	6.02E-05	7.04E-06	10.314	1	PBP-CVD-REC66-03022022	PBP-CVD-U-Rx17-03022022	Kinesin-like protein KIF21B
O95568	METTL18	2	1	13.671	1.51E-06	2.96E-07	13.072	1	PBP-CVD-REC66-03022022	PBP-CVD-HC22-03022022	Histidine protein methyltransferase 1 homolog
P0CF74	IGLC6	7	1	90.275	4.24E-03	3.05E-04	2.925	0.949	PBP-CVD-REC66-03022022	PBP-CVD-U-Rx17-03022022	Immunoglobulin lambda constant 6
P0DOX2	Immunoglob a-2	21	5	224.882	3.09E-07	7.43E-08	6.204	1	PBP-CVD-REC66-03022022	PBP-CVD-U-Rx17-03022022	Immunoglobulin alpha-2 heavy chain
P0DP06	IGHV4-30-4	4	2	43.006	1.20E-03	9.56E-05	3.001	0.988	PBP-CVD-REC66-03022022	PBP-CVD-MOD15-03022022	Immunoglobulin heavy variable 4-30-4
P00742	F10	5	5	35.978	3.70E-04	3.39E-05	3.327	0.999	PBP-CVD-REC66-03022022	PBP-CVD-U-Rx17-03022022	Coagulation factor X
P00915	CA1	3	3	16.470	9.28E-09	4.01E-09	5.262	1	PBP-CVD-REC66-03022022	PBP-CVD-HC22-03022022	Carbonic anhydrase 1
P01599	IGKV1-17	2	1	12.987	1.53E-05	2.17E-06	287.440	1	PBP-CVD-REC66-03022022	PBP-CVD-HC22-03022022	Immunoglobulin kappa variable 1-17
P01602	IGKV1-5	4	2	46.209	2.60E-06	4.74E-07	6.595	1	PBP-CVD-REC66-03022022	PBP-CVD-MOD15-03022022	Immunoglobulin kappa variable 1-5
P01701	IGLV1-51	3	3	17.733	2.32E-06	4.34E-07	4.630	1	PBP-CVD-REC66-03022022	PBP-CVD-MOD15-03022022	Immunoglobulin lambda variable 1-51
P01717	IGLV3-25	3	1	29.484	1.02E-06	2.11E-07	4.458	1	PBP-CVD-REC66-03022022	PBP-CVD-U-Rx17-03022022	Immunoglobulin lambda variable 3-25
P01859	IGHG2	18	8	238.787	4.51E-04	3.99E-05	5.044	0.997	PBP-CVD-REC66-03022022	PBP-CVD-HC22-03022022	Immunoglobulin heavy constant gamma 2
P01861	IGHG4	16	6	222.054	1.52E-05	2.16E-06	4.539	1	PBP-CVD-REC66-03022022	PBP-CVD-MOD15-03022022	Immunoglobulin heavy constant gamma 4
P01876	IGHA1	27	17	257.427	3.63E-04	3.34E-05	2.506	0.998	PBP-CVD-REC66-03022022	PBP-CVD-U-Rx17-03022022	Immunoglobulin heavy constant alpha 1
P02008	HBZ	2	1	16.853	4.17E-05	5.02E-06	2.387	1	PBP-CVD-REC66-03022022	PBP-CVD-HC22-03022022	Hemoglobin subunit zeta
P03245	E1B 55 kDa prt	4	2	20.272	7.38E-06	1.15E-06	4.755	1	PBP-CVD-REC66-03022022	PBP-CVD-HC22-03022022	E1B 55 kDa protein

P06312	IGKV4-1	4	4	34.560	2.20E-08	7.92E-09	3.553	1	PBP-CVD-REC66-03022022	PBP-CVD-MOD15-03022022	Immunoglobulin kappa variable 4-1
P07358	C8B	23	20	180.259	4.37E-09	2.31E-09	8.069	1	PBP-CVD-REC66-03022022	PBP-CVD-HC22-03022022	Complement component C8 beta chain
P19793	RXRA	4	3	23.307	2.21E-06	4.20E-07	6.318	1	PBP-CVD-REC66-03022022	PBP-CVD-MOD15-03022022	Retinoic acid receptor RXR-alpha
P22792	CPN2	12	11	111.819	1.68E-04	1.70E-05	2.797	1	PBP-CVD-REC66-03022022	PBP-CVD-U-Rx17-03022022	Carboxypeptidase N subunit 2
P32119	PRDX2	2	2	10.580	1.19E-04	1.25E-05	4.873	1	PBP-CVD-REC66-03022022	PBP-CVD-HC22-03022022	Peroxiredoxin-2
P80748	IGLV3-21	2	2	13.648	6.11E-04	5.29E-05	2.112	0.997	PBP-CVD-REC66-03022022	PBP-CVD-MOD15-03022022	Immunoglobulin lambda variable 3-21
Q5T655	CFAP58	4	3	21.401	7.32E-05	8.42E-06	2.316	1	PBP-CVD-REC66-03022022	PBP-CVD-HC22-03022022	Cilia- and flagella-associated protein 58
Q5VXU9	SHOC1	3	2	19.243	2.31E-04	2.21E-05	10.423	1	PBP-CVD-REC66-03022022	PBP-CVD-MOD15-03022022	Protein shortage in chiasmata 1 ortholog
Q6PG37	ZNF790	4	2	17.563	2.27E-02	1.43E-03	5.783	0.781	PBP-CVD-REC66-03022022	PBP-CVD-MOD15-03022022	Zinc finger protein 790
Q6Q4G3	LVRN	3	2	27.515	5.10E-06	8.42E-07	2.648	1	PBP-CVD-REC66-03022022	PBP-CVD-U-Rx17-03022022	Aminopeptidase Q
Q7Z698	SPRED2	2	2	17.922	4.07E-02	2.44E-03	19.819	0.601	PBP-CVD-REC66-03022022	PBP-CVD-HC22-03022022	Sprouty-related_ EVH1 domain-containing protein 2
Q8N4C6	NIN	4	2	26.216	1.59E-02	1.03E-03	2.846	0.841	PBP-CVD-REC66-03022022	PBP-CVD-MOD15-03022022	Ninein
Q8N335	GPD1L	3	2	18.931	5.88E-07	1.31E-07	2.716	1	PBP-CVD-REC66-03022022	PBP-CVD-U-Rx17-03022022	Glycerol-3 phosphate dehydrogenase 1-like protein
Q8WXW3	PIBF1	9	7	63.457	7.48E-05	8.49E-06	2.341	1	PBP-CVD-REC66-03022022	PBP-CVD-U-Rx17-03022022	Progesterone-induced-blocking factor 1
Q9BXF6	RAB11FIP5	5	4	36.221	2.88E-07	7.08E-08	4.723	1	PBP-CVD-REC66-03022022	PBP-CVD-HC22-03022022	Rab11 family-interacting protein 5
Q9BXU0	TEX12	2	2	17.584	4.00E-05	4.89E-06	8.046	1	PBP-CVD-REC66-03022022	PBP-CVD-MOD15-03022022	Testis-expressed protein 12

Q9NRF2	SH2B1	4	3	29.570	2.13E-03	1.63E-04	3.942	0.969	PBP-CVD-REC66-03022022	PBP-CVD-MOD15-03022022	SH2B adapter protein 1
Q9NY93	DDX56	4	4	37.805	7.92E-06	1.22E-06	9.671	1	PBP-CVD-REC66-03022022	PBP-CVD-MOD15-03022022	Probable ATP-dependent RNA helicase DDX56
Q9NYR8	RDH8	2	1	11.458	1.20E-07	3.65E-08	536.662	1	PBP-CVD-REC66-03022022	PBP-CVD-HC22-03022022	Retinol dehydrogenase 8
Q9UHR6	ZNHIT2	9	7	53.631	9.15E-04	7.56E-05	2.014	0.989	PBP-CVD-REC66-03022022	PBP-CVD-HC22-03022022	Zinc finger HIT domain-containing protein 2
Q9UPW6	SATB2	2	1	11.584	3.83E-11	8.13E-11	27013.782	1	PBP-CVD-REC66-03022022	PBP-CVD-HC22-03022022	DNA-binding protein SATB2
Q9Y2H2	INPP5F	5	1	35.138	4.08E-06	7.06E-07	17.126	1	PBP-CVD-REC66-03022022	PBP-CVD-HC22-03022022	Phosphatidylinositi de phosphatase SAC2
Q9Y2L1	DIS3	3	1	16.180	2.41E-02	1.52E-03	3.933	0.735	PBP-CVD-REC66-03022022	PBP-CVD-U-Rx17-03022022	Exosome complex exonuclease RRP44
Q9Y2X9	ZNF281	2	1	17.020	1.98E-02	1.26E-03	28.308	0.745	PBP-CVD-REC66-03022022	PBP-CVD-HC22-03022022	Zinc finger protein 281
Q86T90	KIAA1328	4	3	28.831	7.10E-04	5.99E-05	4.502	0.996	PBP-CVD-REC66-03022022	PBP-CVD-MOD15-03022022	Protein hinderin
Q86W28	NLRP8	9	6	58.492	3.23E-09	1.84E-09	5.243	1	PBP-CVD-REC66-03022022	PBP-CVD-HC22-03022022	NACHT_ LRR and PYD domains-containing protein 8
Q96A32	MYL11	2	1	11.471	7.25E-07	1.55E-07	Infinity	1	PBP-CVD-REC66-03022022	PBP-CVD-HC22-03022022	Myosin regulatory light chain 2_ skeletal muscle isoform
Q02985	CFHR3	12	7	75.537	6.96E-04	5.90E-05	2.062	0.991	PBP-CVD-REC66-03022022	PBP-CVD-HC22-03022022	Complement factor H-related protein 3
Q15020	SART3	5	3	45.580	6.60E-04	5.65E-05	5.809	0.997	PBP-CVD-REC66-03022022	PBP-CVD-U-Rx17-03022022	Squamous cell carcinoma antigen recognized by T-cells 3
A0A0A0MRZ7	IGKV2D-26	6	3	20.872	1.10E-04	1.18E-05	8.121	1	PBP-CVD-U-Rx17-03022022	PBP-CVD-MOD15-03022022	Immunoglobulin kappa variable 2D-26
A0A0B4J1X5	IGHV3-74	4	1	39.747	1.30E-05	1.90E-06	3.984	1	PBP-CVD-U-Rx17-03022022	PBP-CVD-MOD15-03022022	Immunoglobulin heavy variable 3-74

A3KMH1	VWA8	15	10	100.861	2.99E-04	2.78E-05	5.053	0.999	PBP-CVD-U-Rx17-03022022	PBP-CVD-HC22-03022022	von Willebrand factor A domain-containing protein 8
E7EW31	PROB1	9	7	52.623	2.11E-07	5.61E-08	8.117	1	PBP-CVD-U-Rx17-03022022	PBP-CVD-HC22-03022022	Proline-rich basic protein 1
O00750	PIK3C2B	4	2	28.916	3.62E-10	4.45E-10	31.829	1	PBP-CVD-U-Rx17-03022022	PBP-CVD-HC22-03022022	Phosphatidylinositol 4-phosphate 3-kinase C2 domain-containing subunit beta
O14531	DPYSL4	2	1	12.041	1.52E-06	2.96E-07	Infinity	1	PBP-CVD-U-Rx17-03022022	PBP-CVD-HC22-03022022	Dihydropyrimidinase-related protein 4
O15085	ARHGEF11	3	2	17.026	9.15E-05	1.01E-05	2.625	1	PBP-CVD-U-Rx17-03022022	PBP-CVD-HC22-03022022	Rho guanine nucleotide exchange factor 11
O60476	MAN1A2	5	4	28.599	3.13E-10	4.07E-10	11.849	1	PBP-CVD-U-Rx17-03022022	PBP-CVD-HC22-03022022	Mannosyl-oligosaccharide 1_2-alpha-mannosidase IB
P0DP01	IGHV1-8	3	2	17.170	8.71E-07	1.83E-07	3.974	1	PBP-CVD-U-Rx17-03022022	PBP-CVD-HC22-03022022	Immunoglobulin heavy variable 1-8
P00739	HPR	39	19	305.562	5.12E-06	8.42E-07	3.000	1	PBP-CVD-U-Rx17-03022022	PBP-CVD-HC22-03022022	Haptoglobin-related protein
P01008	SERPINC1	35	35	295.041	4.91E-06	8.25E-07	2.072	1	PBP-CVD-U-Rx17-03022022	PBP-CVD-REC66-03022022	Antithrombin-III
P01767	IGHV3-53	5	2	52.645	3.90E-09	2.12E-09	7.838	1	PBP-CVD-U-Rx17-03022022	PBP-CVD-HC22-03022022	Immunoglobulin heavy variable 3-53
P02649	APOE	24	23	253.763	1.39E-05	2.00E-06	2.543	1	PBP-CVD-U-Rx17-03022022	PBP-CVD-REC66-03022022	Apolipoprotein E
P02654	APOC1	4	4	36.352	4.96E-08	1.65E-08	5.814	1	PBP-CVD-U-Rx17-03022022	PBP-CVD-REC66-03022022	Apolipoprotein C-I
P02786	TFRC	2	1	11.894	2.87E-09	1.76E-09	18.925	1	PBP-CVD-U-Rx17-03022022	PBP-CVD-HC22-03022022	Transferrin receptor protein 1
P03952	KLKB1	26	22	198.882	4.44E-09	2.31E-09	5.622	1	PBP-CVD-U-Rx17-03022022	PBP-CVD-HC22-03022022	Plasma kallikrein
P04070	PROC	5	5	29.386	3.04E-05	3.90E-06	3.000	1	PBP-CVD-U-Rx17-03022022	PBP-CVD-REC66-03022022	Vitamin K-dependent protein C
P04180	LCAT	3	2	17.008	2.13E-05	2.86E-06	2.389	1	PBP-CVD-U-Rx17-03022022	PBP-CVD-HC22-03022022	Phosphatidylcholine-sterol acyltransferase

P06396	GSN	34	33	287.598	9.10E-06	1.38E-06	3.135	1	PBP-CVD-U-Rx17-03022022	PBP-CVD-HC22-03022022	Gelsolin
P07357	C8A	17	17	131.123	1.16E-05	1.72E-06	2.450	1	PBP-CVD-U-Rx17-03022022	PBP-CVD-REC66-03022022	Complement component C8 alpha chain
P07988	SFTPB	4	3	29.683	7.89E-06	1.22E-06	2.891	1	PBP-CVD-U-Rx17-03022022	PBP-CVD-REC66-03022022	Pulmonary surfactant-associated protein B
P11226	MBL2	2	2	10.465	2.97E-07	7.23E-08	80.707	1	PBP-CVD-U-Rx17-03022022	PBP-CVD-HC22-03022022	Mannose-binding protein C
P14350	pol	3	3	19.756	9.16E-06	1.38E-06	3.193	1	PBP-CVD-U-Rx17-03022022	PBP-CVD-HC22-03022022	Pro-Pol polyprotein
P17936	IGFBP3	4	2	27.709	5.30E-07	1.21E-07	87.058	1	PBP-CVD-U-Rx17-03022022	PBP-CVD-MOD15-03022022	Insulin-like growth factor-binding protein 3
P27918	CFP	3	3	23.164	6.64E-03	4.61E-04	4.983	0.946	PBP-CVD-U-Rx17-03022022	PBP-CVD-MOD15-03022022	Properdin
P35858	IGFALS	18	16	147.995	6.93E-05	8.01E-06	2.378	1	PBP-CVD-U-Rx17-03022022	PBP-CVD-MOD15-03022022	Insulin-like growth factor-binding protein complex acid labile subunit
P35916	FLT4	3	2	16.680	5.39E-09	2.68E-09	66.381	1	PBP-CVD-U-Rx17-03022022	PBP-CVD-HC22-03022022	Vascular endothelial growth factor receptor 3
P35968	KDR	4	3	27.673	4.26E-05	5.11E-06	2.959	1	PBP-CVD-U-Rx17-03022022	PBP-CVD-HC22-03022022	Vascular endothelial growth factor receptor 2
P49411	TUFM	6	2	38.460	1.44E-09	1.16E-09	14.815	1	PBP-CVD-U-Rx17-03022022	PBP-CVD-MOD15-03022022	Elongation factor Tu_ mitochondrial
P51530	DNA2	5	3	32.369	5.63E-06	9.07E-07	2.111	1	PBP-CVD-U-Rx17-03022022	PBP-CVD-HC22-03022022	DNA replication ATP-dependent helicase/ nuclease DNA2
P51805	PLXNA3	12	9	73.644	2.30E-05	3.04E-06	2.655	1	PBP-CVD-U-Rx17-03022022	PBP-CVD-HC22-03022022	Plexin-A3
P53675	CLTCL1	4	3	21.137	9.94E-07	2.07E-07	198.440	1	PBP-CVD-U-Rx17-03022022	PBP-CVD-HC22-03022022	Clathrin heavy chain 2
P62736	ACTA2	6	3	31.470	4.20E-08	1.44E-08	11.432	1	PBP-CVD-U-Rx17-03022022	PBP-CVD-HC22-03022022	Actin_ aortic smooth muscle

Q2M1P5	KIF7	8	3	49.869	1.17E-08	4.81E-09	5.474	1	PBP-CVD-U-Rx17-03022022	PBP-CVD-HC22-03022022	Kinesin-like protein KIF7
Q4V328	GRIPAP1	3	2	23.986	3.27E-05	4.09E-06	4.972	1	PBP-CVD-U-Rx17-03022022	PBP-CVD-MOD15-03022022	GRIP1-associated protein 1
Q6Q0C0	TRAF7	4	3	23.295	9.87E-04	8.01E-05	3.489	0.995	PBP-CVD-U-Rx17-03022022	PBP-CVD-HC22-03022022	E3 ubiquitin-protein ligase TRAF7
Q6UWW8	CES3	2	1	11.913	7.63E-04	6.42E-05	68.405	0.994	PBP-CVD-U-Rx17-03022022	PBP-CVD-HC22-03022022	Carboxylesterase 3
Q6ZMP0	THSD4	5	2	26.608	1.39E-03	1.09E-04	76.538	0.988	PBP-CVD-U-Rx17-03022022	PBP-CVD-HC22-03022022	Thrombospondin type-1 domain-containing protein 4
Q6ZMT9	DTHD1	3	1	17.401	2.65E-09	1.68E-09	13459.116	1	PBP-CVD-U-Rx17-03022022	PBP-CVD-REC66-03022022	Death domain-containing protein 1
Q6ZRR7	LRRC9	4	2	28.787	6.97E-04	5.90E-05	3.026	0.998	PBP-CVD-U-Rx17-03022022	PBP-CVD-HC22-03022022	Leucine-rich repeat-containing protein 9
Q8N9V7	TOPAZ1	11	8	77.297	1.90E-05	2.62E-06	2.043	1	PBP-CVD-U-Rx17-03022022	PBP-CVD-REC66-03022022	Protein TOPAZ1
Q8WVF5	KCTD4	4	4	19.344	3.69E-07	8.71E-08	294.345	1	PBP-CVD-U-Rx17-03022022	PBP-CVD-HC22-03022022	BTB/POZ domain-containing protein KCTD4
Q8WZ64	ARAP2	9	7	49.989	3.26E-05	4.09E-06	8.793	1	PBP-CVD-U-Rx17-03022022	PBP-CVD-HC22-03022022	Arf-GAP with Rho-GAP domain_ ANK repeat and PH domain-containing protein 2
Q9BRJ2	MRPL45	3	3	26.377	4.68E-03	3.34E-04	2.415	0.936	PBP-CVD-U-Rx17-03022022	PBP-CVD-HC22-03022022	39S ribosomal protein L45_ mitochondrial
Q9BTE7	DCUN1D5	4	3	27.243	8.78E-12	3.11E-11	58.318	1	PBP-CVD-U-Rx17-03022022	PBP-CVD-HC22-03022022	DCN1-like protein 5
Q9HC38	GLOD4	2	2	17.331	2.34E-03	1.75E-04	2.412	0.976	PBP-CVD-U-Rx17-03022022	PBP-CVD-REC66-03022022	Glyoxalase domain-containing protein 4
Q9NWZ8	GEMIN8	2	2	17.152	8.18E-09	3.67E-09	11.902	1	PBP-CVD-U-Rx17-03022022	PBP-CVD-HC22-03022022	Gem-associated protein 8
Q9UFE4	CCDC39	3	2	18.089	7.21E-07	1.55E-07	15.061	1	PBP-CVD-U-Rx17-03022022	PBP-CVD-HC22-03022022	Coiled-coil domain-containing protein 39
Q9UFN0	NIPSNAP3A	3	2	23.384	1.05E-10	1.88E-10	15.819	1	PBP-CVD-U-Rx17-03022022	PBP-CVD-HC22-03022022	Protein NipSnap homolog 3A

Q9UK55	SERPINA10	13	11	89.264	1.99E-05	2.72E-06	8.883	1	PBP-CVD-U-Rx17-03022022	PBP-CVD-HC22-03022022	Protein Z-dependent protease inhibitor
Q9ULH1	ASAP1	8	4	53.492	7.05E-06	1.11E-06	3.728	1	PBP-CVD-U-Rx17-03022022	PBP-CVD-HC22-03022022	Arf-GAP with SH3 domain_ ANK repeat and PH domain-containing protein 1
Q9Y4F3	MARF1	4	2	21.955	6.36E-07	1.39E-07	28.725	1	PBP-CVD-U-Rx17-03022022	PBP-CVD-HC22-03022022	Meiosis regulator and mRNA stability factor 1
Q9Y6D0	SELENOK	2	1	18.573	1.78E-07	5.02E-08	2.783	1	PBP-CVD-U-Rx17-03022022	PBP-CVD-HC22-03022022	Selenoprotein K
Q9Y6N7	ROBO1	8	5	58.651	7.63E-10	7.43E-10	39.874	1	PBP-CVD-U-Rx17-03022022	PBP-CVD-HC22-03022022	Roundabout homolog 1
Q30KQ8	DEFB112	2	2	10.425	4.62E-04	4.07E-05	2.643	0.999	PBP-CVD-U-Rx17-03022022	PBP-CVD-REC66-03022022	Beta-defensin 112
Q86TI0	TBC1D1	3	2	16.249	1.31E-06	2.65E-07	13.384	1	PBP-CVD-U-Rx17-03022022	PBP-CVD-HC22-03022022	TBC1 domain family member 1
Q96DY7	MTBP	6	4	33.440	3.81E-04	3.48E-05	2.556	1	PBP-CVD-U-Rx17-03022022	PBP-CVD-MOD15-03022022	Mdm2-binding protein
Q96IY4	CPB2	7	6	50.503	5.68E-07	1.28E-07	5.724	1	PBP-CVD-U-Rx17-03022022	PBP-CVD-HC22-03022022	Carboxypeptidase B2
Q02045	MYL5	2	1	10.499	1.85E-09	1.39E-09	3.267	1	PBP-CVD-U-Rx17-03022022	PBP-CVD-HC22-03022022	Myosin light chain 5
Q02809	PLOD1	2	1	11.881	1.16E-09	1.04E-09	317.549	1	PBP-CVD-U-Rx17-03022022	PBP-CVD-HC22-03022022	Procollagen-lysine_2-oxoglutarate 5-dioxygenase 1
Q13367	AP3B2	2	1	12.927	9.12E-03	6.12E-04	13.757	0.863	PBP-CVD-U-Rx17-03022022	PBP-CVD-HC22-03022022	AP-3 complex subunit beta-2
Q13976	PRKG1	3	2	18.029	1.54E-07	4.50E-08	11.720	1	PBP-CVD-U-Rx17-03022022	PBP-CVD-REC66-03022022	cGMP-dependent protein kinase 1
Q14696	MESD	3	3	21.021	6.94E-12	3.11E-11	135.864	1	PBP-CVD-U-Rx17-03022022	PBP-CVD-HC22-03022022	LRP chaperone MESD
Q15166	PON3	3	1	35.480	9.13E-05	1.01E-05	4.758	1	PBP-CVD-U-Rx17-03022022	PBP-CVD-HC22-03022022	Serum paraoxonase/lactonase 3
Q16623	STX1A	2	2	10.110	3.21E-05	4.07E-06	13.371	1	PBP-CVD-U-Rx17-03022022	PBP-CVD-HC22-03022022	Syntaxin-1A

Q16880	UGT8	5	4	38.893	5.90E-05	6.93E-06	5.604	1	PBP-CVD-U-Rx17-03022022	PBP-CVD-HC22-03022022	2-hydroxy-acylsphingosine 1-beta-galactosyl-transferase

HC = healthy, MOD = moderate, RX = under medication, REC = recovered
